# Long-term Survival of Hip Cement Spacer: A Case Report

**DOI:** 10.1055/s-0041-1736341

**Published:** 2021-11-11

**Authors:** Roshan Jacob, Mackenzie Sowers, Kelly Chandler, Mihir Patel, Ashish B. Shah, Sameer Mahadeorao Naranje

**Affiliations:** 1 Departamento de Cirurgia Ortopédica, University of Alabama at Birmingham, Birmingham, Alabama, Estados Unidos

**Keywords:** arthroplasty, replacement, hip, bone cements, hip joint, hip prosthesis, prosthesis-related infections

## Abstract

We present a unique case of a 56-year-old male patient who ambulated on a hip cement spacer for 11 years. After hemiarthroplasty after a motor vehicle accident, the patient developed periprosthetic joint infection (PJI) several years later, and underwent stage-1 revision. With the resolution of the infection after stage 1, the patient refused the second stage due to satisfaction with the cement spacer for nearly 11 years.

To our knowledge, this is the longest reported case of a cement spacer remaining in an ambulating patient. This case demonstrates the mechanical reliability of metal-reinforced cement spacers, which can remain for long periods in selected patients.

## Introduction

While total hip arthroplasty (THA) is considered one of the most performed and successful elective surgeries in the United States, periprosthetic joint infection (PJI) is a devastating complication due to the reoperation and readmission necessary to treat the infection. 1 While the most frequently used technique to treat infected hip arthroplasty in the United States is two-stage revision using antibiotic-impregnated cement with an antibiotic spacer, the management of PJI and the treatment options should be individualized according to the needs of each patient. 2

The purpose of the antibiotic spacer is to provide a higher concentration of medication directly around the area of infection, and this is achieved by placing a drug-eluting femoral stem with antibiotic-impregnated cement. 2 This is intended as a temporary measure, as cement hip spacers have been reported to have a dislocation rate of 7% and a fracture rate of 2%. 2 However, newer, prefabricated metal-reinforced spacers have been shown to have much higher mechanical strength, with spacer fracture reports as low as 0% in several studies. 3 4 5 6 The goal of reporting the present case is to describe and discuss the long-term results of an articulating hip spacer placed due to PJI, after which the patient continued to have full function of his hip for over 11 years before the second-stage revision. To our knowledge, this is the longest reported case of a retained spacer in a patient being treated for PJI.

## Case Report

**Fig. 1:**
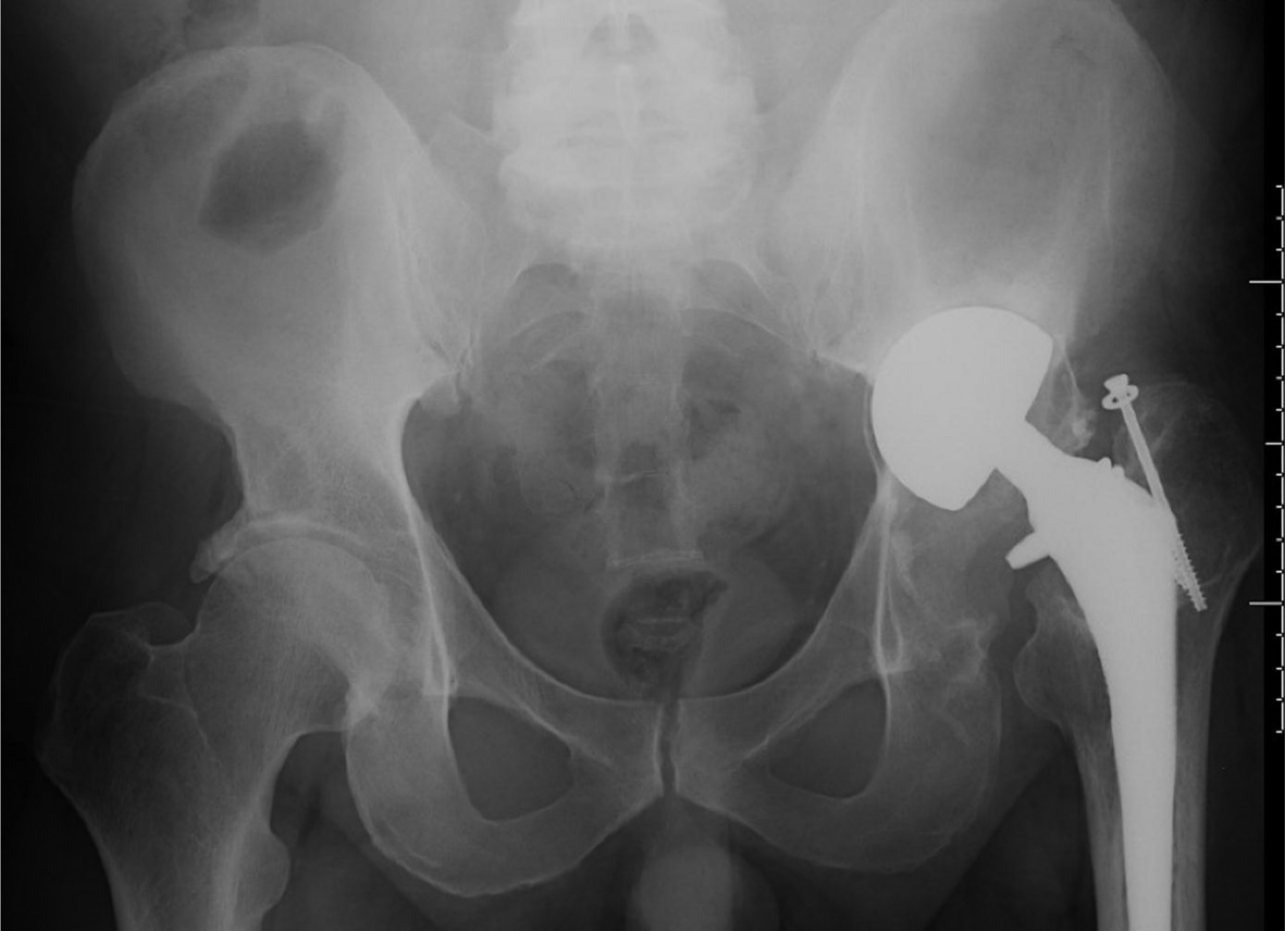
Anteroposterior radiograph of the pelvis showing a hemiarthroplasty 14 years after surgery.

A 56-year-old morbidly obese male patient with a history of left hip fracture and hemiarthroplasty who presented with new hip pain. He had been involved in a motor vehicle collision (MVC) 25 years prior, and had undergone left hip hemiarthroplasty at another institution ( Fig. 1 ). The patient lived with pain for 14 years until he developed signs of PJI, presenting with elevated erythrocyte sedimentation rate (ESR, 36 mm/h) and elevated C reactive protein (CRP, 25 mg/L), and eventual sinus and drainage. After discussion, the patient agreed to undergo two-stage hip revision.

**Fig. 2:**
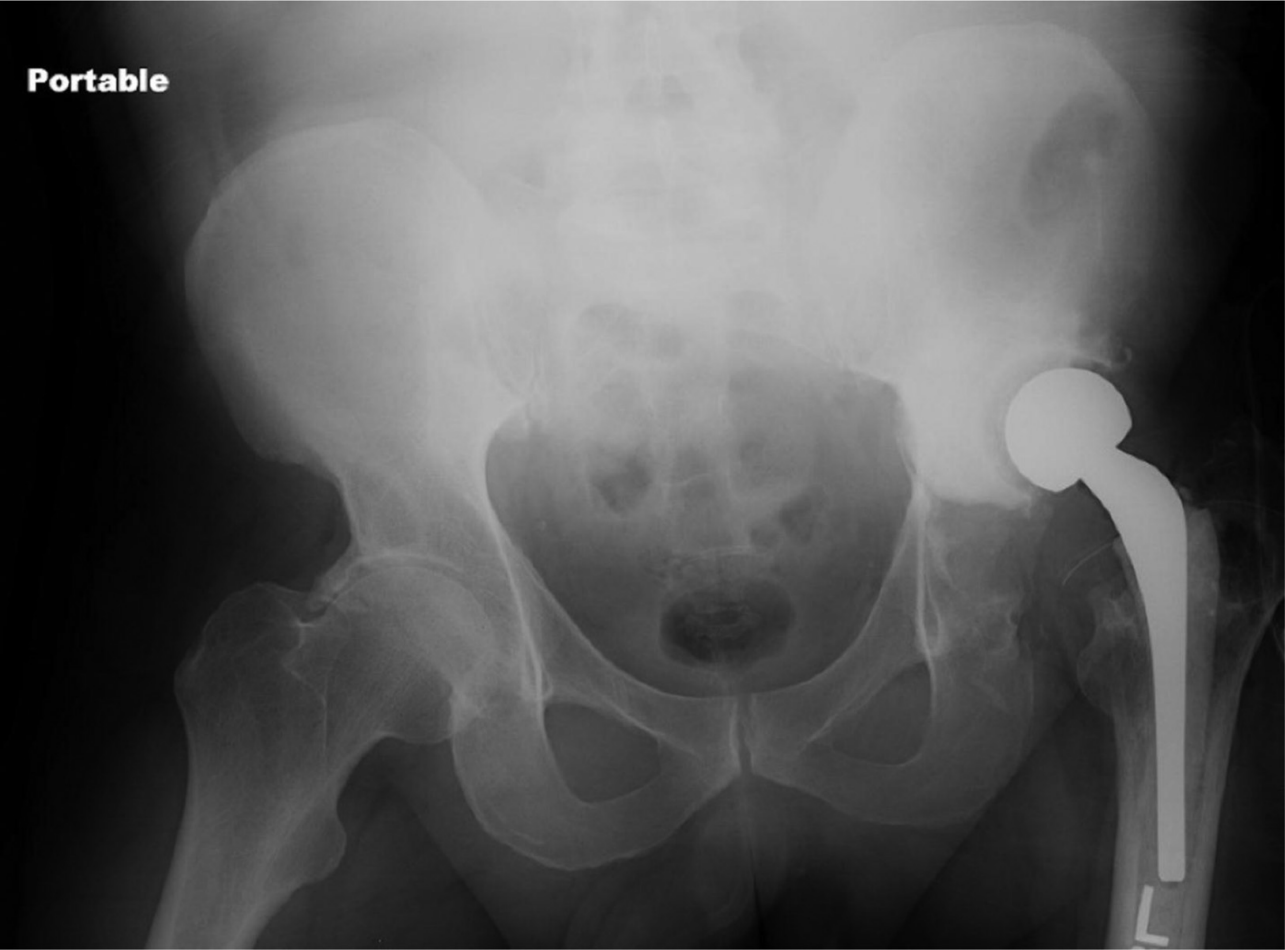
Anteroposterior radiograph of the pelvis showing a Prostalac spacer immediately following stage-1 revision.

Using a posterior approach, an antibiotic cement and polytheline liner were placed without reaming on the acetabular side after cleaning and debriding it. Extraction of the femoral stem and head was performed with no bone loss, and the femoral canal was reamed and cleared of debris distally. After irrigation and debridement, the femoral canal was broached, and a size 3 Prostalac (Depuy-Synthes, Warsaw, IN, US) antibiotic cement stem and appropriately-sized head was placed. After hip reduction, a Hemovac drain (Zimmer Biomet, Warsaw, IN, US) was placed, and the wound was closed in a standard manner. There were no surgical complications ( Fig. 2 ). A microbiology report of fluid taken during surgery revealed *Staphylococcus lugdunensis* with no organisms isolated from blood samples.

**Fig. 3:**
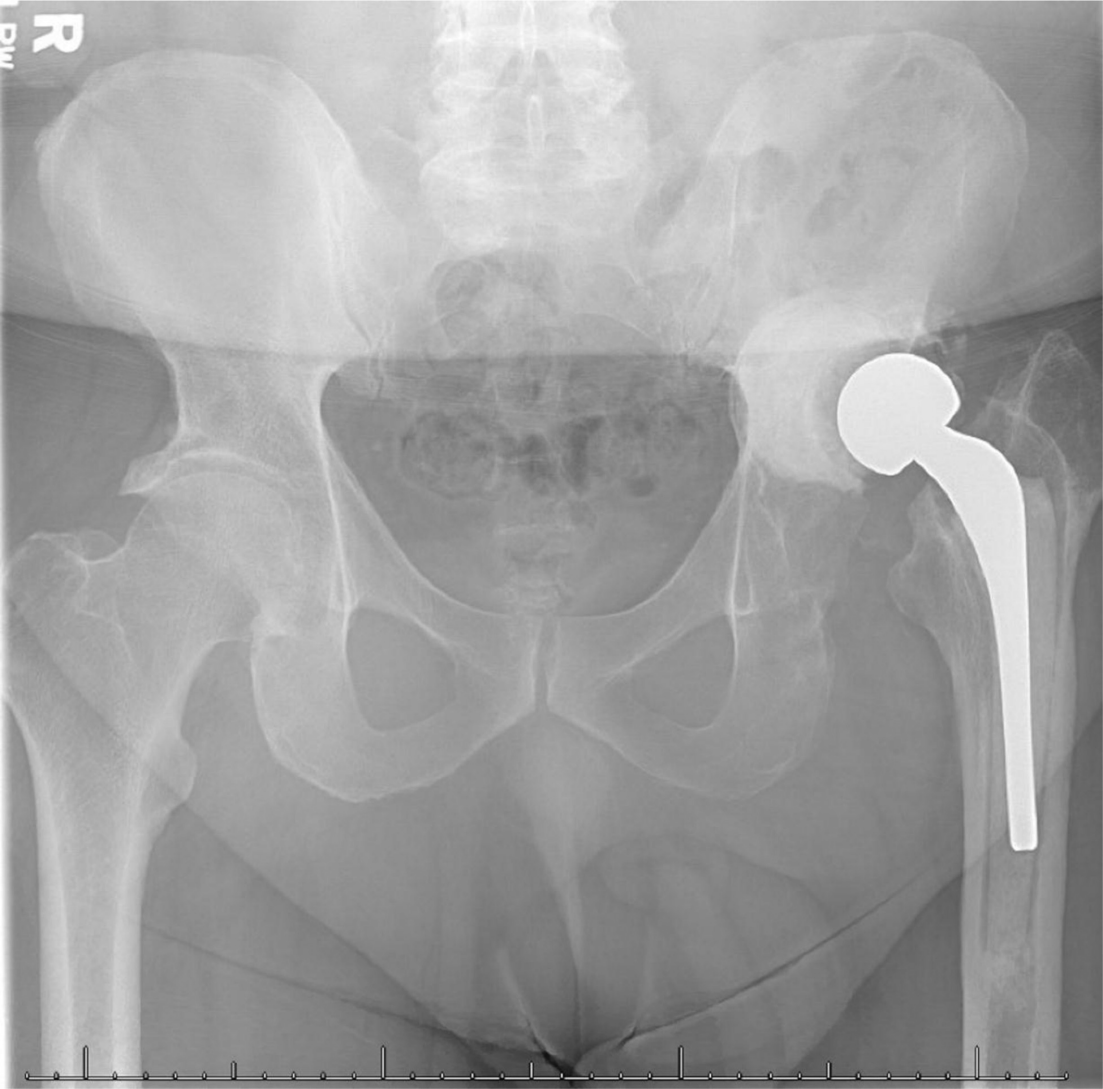
Anteroposterior radiograph of the pelvis showing a Prostalac spacer retained for 11 years.

The patient experienced gradual resolution of the infection and pain postoperatively after the appropriate antibiotic treatment. Over the following years, he continued his daily activities, including hunting and fishing. The patient refused the second stage of the revision as he was satisfied with the spacer. Eleven years later, he returned to the clinic complaining of pain in his left hip. He did not complain of any drainage or fevers. An imaging exam revealed loosening of the femoral component, and the patient agreed to second-stage revision of the THA ( Fig. 3 ). ESR (5 mm/h) and CRP (1.62 mg/L) returned to within normal limits before spacer removal, with no elevation in the white-cell count, satisfying the criteria by Parvizi et al. 7 for absence of PJI.

**Fig. 4:**
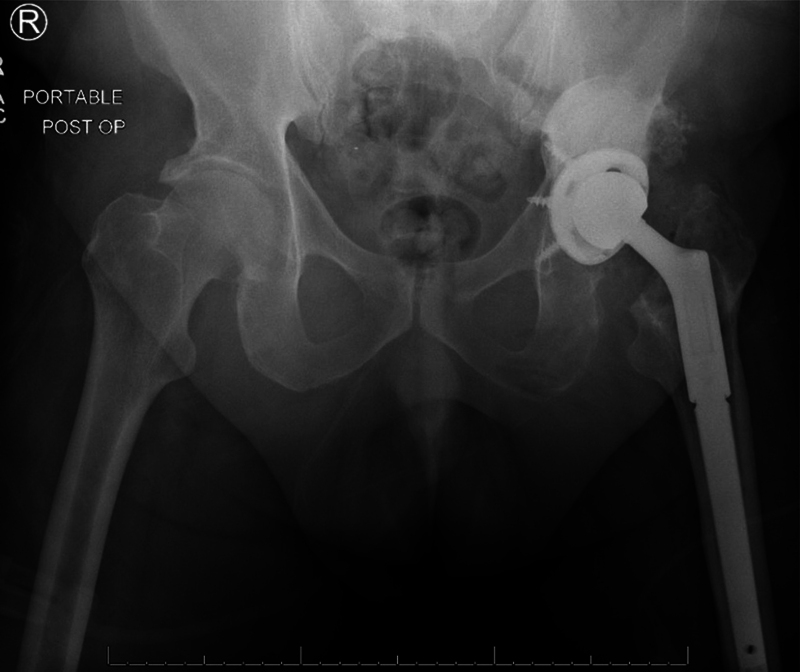
Anteroposterior radiograph of the pelvis showing a total hip arthroplasty as stage-2 revision.

A classic posterior approach was used. The stem and liner were loose and removed uneventfully. There was no evidence of bone loss (type I in the Paprosky classification). The acetabulum was reamed, and an appropriately-sized revision cup was secured with multiple screws. The remaining bone deficit was filled with antibiotic cement. The femur was prepared using the standard technique for modular revision stem. An appropriately-sized modular stem and metal head were placed and easily implanted eith the use of the standard technique after initially performing a trial reduction. The hip reduced, and the wound was closed using the standard technique. The microbiology reports showed no organism growth from the cultures obtained intraoperatively. Upon his most recent follow-up, three months postoperatively, the patient was ambulating with no pain, no drainage from his operated hip, and no discrepancy regarding leg length ( Fig. 4 ).

Informed consent was obtained from the patient whose case is herein reported.

## Discussion

While two-stage revision is the preferred technique for the management of PJI of the hip, the cement hip spacer is not considered the definitive implant in terms of long-term survival. These spacers are intended for short-term use only, until the infection is controlled. However, in the present case report, we discuss how a temporary cement hip spacer was tolerated by the patient over a long period of time (eleven years).

Mechanical strength and fixation are the greatest weak points of the cement hip spacer. A study 8 involving 35 patients with a median age of 56, with surgeon-made articulating polymethylmethacrylate (PMMA) spacers, showed a mechanical failure rate (subsidence or fracture) of 45% ( *n* = 14), with an average period of 14.5 weeks between procedures. Spacer dislocation occurred in 19% of the patients ( *n* = 6). The authors also found that failure rates were significantly higher in younger individuals due to the higher demand placed on the spacer. 8 Recently, surgeons have moved to using prefabricated spacers instead of surgeon-made PMMA spacers due to the superior results. 3 4 8 Prostalac spacers in particular have shown excellent mechanical stability in several studies, with several reports of these spacers maintaining stability for up to 6 years. 5 6 9 10 11 The patient presented here had a Prostalac stem and a polyethylene liner that maintained mechanical stability, and the patient maintained mobility for up to 11 years on the stem, without signs of stem fracture or dislocation.

In a retrospective study 11 on unplanned retained spacers, the authors reviewed 11 hips that had undergone stage 1 of the revision. Of these patients, 9 remained free of complications, while 1 patient experienced reinfection at 24 months, and the second patient developed aseptic subsidence at 72 months. The mean follow-up for the remaining 9 patients was of 48 months, during which they had no spacer-related complaints and wished to delay surgery. 11

In a separate study 6 following 25 patients with 2-year follow-up for Prostalac spacer revision, the authors found no spacer-related complications, with 68% of the patients not having undergone reimplantation at 24 months. The patients that did undergo revision in this period were younger, more physically demanding patients. It appears that a large proportion of patients can remain with a spacer for several years without serious complications. Given that many of these patients have comorbidities and contraindications to surgery, this gives both the patient and surgeon an alternate to reimplantation in high-risk cases.

To our knowledge, this is the longest reported case of a retained, prefabricated hip spacer for PJI. We suggest that these spacers can remain in selected patients with good functional long-term results. This is beneficial, as it gives surgeons a wider scope of options when it comes to treating higher-risk patients who are not suitable or unwilling to have the second-stage procedure.
