# Supplementary material for: Long-term Survival of Hip Cement Spacer: A Case Report
Source: Rev Bras Ortop (Sao Paulo). 2021 Nov 11;59(5):e793–6. doi: 10.1055/s-0041-1736341 (PMC11624930; doi:10.1055/s-0041-1736341)
Supplement: Related texts

# Research article: Sobrevida em longo prazo do espaçador cimentado para quadril: Relato de caso

**Keywords:** articulação do quadril, artroplastia de quadril, cimentos ósseos, infecções relacionadas às próteses, prótese de quadril

## Abstract

Apresentamos um caso único de um homem de 56 anos que deambulou com um espaçador cimentado para quadril durante 11 anos. Após ser submetido a uma hemiartroplastia em virtude de um acidente de carro, o paciente desenvolveu uma infecção articular periprotética (IAP) vários anos depois, sendo submetido à técnica de revisão da etapa 1. Com a resolução da infecção depois da etapa 1, o paciente recusou a segunda etapa, devido à satisfação com o espaçador cimentado por quase 11 anos.

Pelo que sabemos, este é o caso mais longo relatado de um espaçador cimentado em um paciente deambulando. Este caso demonstra a confiabilidade mecânica dos espaçadores cimentados reforçados com metal que podem ser mantidos por um prazo longo em pacientes selecionados.

## Introdução

Embora a artroplastia total do quadril (ATQ) seja uma das cirurgias eletivas mais realizadas e bem-sucedidas nos Estados Unidos, a infecção articular periprotética (IAP) é uma complicação devastadora, devido à necessidade de reoperação e reinternação para tratá-la. 1 Nos Estados Unidos, a técnica mais frequentemente usada para o tratamento da artroplastia de quadril infectada é a revisão em duas etapas utilizando o espaçador cimentado com antibiótico. O manejo da IAP, assim como as opções de tratamento, devem ser individualizadas de acordo com as necessidades de cada paciente. 2

O objetivo do espaçador com antibiótico é fornecer uma concentração mais elevada do fármaco diretamente ao redor da área de infecção, que é alcançada com a colocação de uma haste femoral farmacológica com cimento ortopédico injetado com antibiótico. 2 Isso é uma medida temporária, pois os espaçadores de quadril cimentado apresentam uma taxa de deslocamento de 7% e uma taxa de fratura de 2%. 2 No entanto, os espaçadores mais novos, pré-moldados e reforçados com metal, mostraram ter uma resistência mecânica muito maior, com relatos de fratura do espaçador da ordem de 0%. 3 4 5 6 O objetivo de divulgar este relato de caso é apresentar e discutir os resultados no longo prazo de um espaçador articular de quadril colocado em virtude de IAP. Após a colocação, o paciente continuou a ter plena função do quadril por mais de 11 anos antes de realizar a revisão da segunda etapa. Pelo que sabemos, este é o caso mais longo relatado de espaçador mantido em um paciente no tratamento de IAP.

## Relato de Caso

Um homem de 56 anos, obeso mórbido, com histórico de fratura do quadril esquerdo e hemiartroplastia, que apresentava um quadro de dor nova no quadril. Ele havia se envolvido em um acidente automobilístico (AA) havia 25 anos, quando foi submetido a hemiartroplastia do quadril esquerdo em outra instituição ( Fig. 1 ). O paciente conviveu com a dor durante 14 anos até desenvolver os sinais da IAP, com a dosagem do exame de velocidade de hemossedimentação (VHS) apresentando um valor de 36 e o de proteína C reativa (PCR), de 25, evoluindo para uma sinusite e drenagem nasal. Com o fracasso do o tratamento intravenoso conservador, o paciente concordou em ser submetido à técnica de revisão do quadril de duas etapas.

**Fig. 1:** Radiografia na incidência anteroposterior (AP) da pelve mostrando hemiartroplastia 14 anos após a cirurgia.

Utilizando uma abordagem posterior, foram colocados o cimento associado ao antibiótico e um forro de polietileno, sem fresagem no lado acetabular após a limpeza e o desbridamento. A extração da haste e da cabeça femorais foi realizada sem perda óssea, sendo o canal femoral fresado e limpo dos detritos distalmente. Após a irrigação e o desbridamento, o canal femoral foi perfurado, e nele foram colocados uma haste de cimento Prostalac (Depuy-Synthes, Warsaw, IN, EUA), tamanho três com antibiótico e uma cabeça de tamanho apropriado. Após a redução do quadril, foi colocado um dreno Hemovac (Zimmer Biomet, Warsaw, IN, EUA), e a ferida, fechada da maneira convencional. Não houve complicações cirúrgicas ( Fig. 2 ). O laudo microbiológico do fluido retirado durante a cirurgia revelou *Staphylococcus lugdunensis* , sem microrganismos isolados a partir das amostras de sangue.

**Fig. 2:** Radiografia na incidência AP da pelve mostrando um espaçador Prostalac imediatamente após a revisão da etapa 1.

No pós-operatório, o paciente apresentou resolução gradual da infecção e da dor com o emprego da antibioticoterapia adequada. Nos anos seguintes, ele prosseguiu com suas atividades diárias, incluindo caça e pesca. O paciente recusou a segunda etapa da revisão por estar satisfeito com o espaçador. Onze anos depois, ele voltou à clínica queixando-se de dores no quadril esquerdo. Ele não reclamou de nenhuma drenagem ou febre. O exame de imagem revelou afrouxamento do componente femoral, tendo o paciente concordado com a revisão de segunda etapa para ATQ ( Fig. 3 ). A dosagem de VHS era 5, e a de PCR, 1,62, antes da remoção do espaçador, sem aumento na contagem dos leucócitos, satisfazendo os critérios de Parvizi et al. 7 para ausência de IAP.

**Fig. 3:** Radiografia na incidência AP da pelve mostrando um espaçador Prostalac retido por 11 anos.

Foi usada uma abordagem posterior clássica. A haste e o revestimento foram soltos e removidos sem intercorrências. Não houve evidência de perda óssea (tipo I na classificação de Paprosky). O acetábulo foi fresado, e uma cúpula de revisão de dimensão adequada foi fixada com vários parafusos. O déficit ósseo remanescente foi preenchido com cimento ortopédico com antibiótico. O fêmur foi preparado usando a técnica padrão para haste de revisão modular. Foram colocadas uma haste modular de tamanho adequado e uma cabeça metálica, que foram implantadas sem qualquer dificuldade com a utilização da técnica convencional depois de se realizar uma redução experimental inicialmente. O quadril foi reduzido, e a ferida, fechada com o uso da técnica convencional. Os laudos microbiológicos não mostraram crescimento de organismos nas culturas obtidas no intraoperatório. No acompanhamento mais recente, três meses após a cirurgia, o paciente deambulava sem dor, sem a utilização de dreno no quadril operado, e sem discrepância no comprimento das pernas ( Fig. 4 ).

**Fig. 4:** Radiografia na incidência AP da pelve mostrando uma artroplastia total do quadril como revisão da etapa 2.

Obteve-se o consentimento informado do paciente cujo caso se relata aqui.

## Discussão

Embora a revisão em duas etapas seja a técnica predileta para o tratamento de IAP do quadril, o espaçador cimentado para quadril não é considerado um implante definitivo com relação à sobrevida em longo prazo. Esses espaçadores destinam-se ao uso de curto prazo, até que a infecção seja controlada. No entanto, neste relato de caso, estamos discutindo como um espaçador temporário cimentado para quadril pode ser tolerado pelo paciente por um longo período (onze anos).

A resistência mecânica e a fixação são os maiores pontos fracos do espaçador cimentado para quadril. Um estudo 8 envolvendo 35 pacientes com idade média de 56 anos, com espaçadores articulados de polimetilmetacrilato (PMMA) feitos por cirurgião, os espaçadores apresentaram uma taxa de falha mecânica (soltura ou fratura) de 45% (n = 14), em um intervalo médio de 14,5 semanas entre os procedimentos. A luxação do espaçador ocorreu em 19% dos pacientes (n = 6). Os autores também observaram que as taxas de falha eram significativamente maiores nos indivíduos mais jovens, em virtude de uma maior demanda imposta ao espaçador. 8 Recentemente, os cirurgiões passaram a usar os espaçadores pré-fabricados, em vez dos espaçadores de PMMA feitos por cirurgiões, devido aos resultados superiores. 3 4 8 Os espaçadores Prostalac, em particular, mostraram excelente estabilidade mecânica em vários estudos, com vários relatos afirmando que os espaçadores mantem a estabilidade por até 6 anos. 5 6 9 10 11 O paciente aqui apresentado era portador de uma haste Prostalac e um forro de polietileno, que manteve a estabilidade mecânica e a mobilidade por até 11 anos, sem que houvesse sinais de fratura ou deslocamento da haste.

Em um estudo retrospectivo 11 em que foi analisada a retenção não intencional dos espaçadores foram reavaliados 11 quadris que haviam sido submetidos à revisão da etapa 1. Desses pacientes, 9 permaneceram sem complicações, ao passo que 1 paciente apresentou reinfecção em 24 meses e o segundo paciente desenvolveu soltura asséptica em 72 meses. O acompanhamento médio dos 9 pacientes restantes foi de 48 meses, durante o quais eles não apresentaram queixas relacionadas ao espaçador, e manifestaram o desejo de adiar a cirurgia. 11

Um outro estudo, 6 com 25 pacientes que tiveram acompanhamento durante 2 anos para a revisão do espaçador Prostalac, não foram observadas complicações relacionadas ao espaçador em 68% dos pacientes, e eles não foram submetidos a reimplante em 24 meses. Os pacientes que foram submetidos a revisão neste período eram mais jovens e mais exigentes fisicamente. Parece que uma grande proporção de pacientes pode permanecer com o espaçador por vários anos sem complicações graves. Visto que muitos desses pacientes apresentam comorbidades e contraindicações à cirurgia, isso oferece ao paciente e ao cirurgião uma alternativa para o reimplante em casos de alto risco.

Pelo que sabemos, este é o caso mais longo relatado de espaçador de quadril pré-moldado retido para IAP. Propomos que esses espaçadores podem permanecer em determinados pacientes com bons resultados funcionais em longo prazo. Isso é benéfico, pois dá aos cirurgiões um leque de opções mais amplo no tocante ao tratamento de pacientes de alto risco que não sejam aptos ou não desejem realizar o procedimento de segunda etapa.
